# Concurrent Validity and Test–Retest Reliability of Pressure-Detecting Insoles for Static and Dynamic Movements in Healthy Young Adults

**DOI:** 10.3390/s23104913

**Published:** 2023-05-19

**Authors:** Johanna Lambrich, Marco Hagen, Gerrit Schwiertz, Thomas Muehlbauer

**Affiliations:** Division of Movement and Training Sciences/Biomechanics of Sport, University of Duisburg-Essen, 45141 Essen, Germany; marco.hagen@uni-due.de (M.H.); gerrit.schwiertz@uni-due.de (G.S.); thomas.muehlbauer@uni-due.de (T.M.)

**Keywords:** standing, walking, running, jumping, pressure insole, force plate, validation, repeatability

## Abstract

Compared to force-plates, pressure-detecting insoles have the advantage that vertical ground reaction force (vGRF) can be estimated under field rather than laboratory conditions. However, the question arises whether insoles also provide valid and reliable results compared to a force-plate (i.e., the gold standard). The study aimed to investigate the concurrent validity and test–retest reliability of pressure-detecting insoles during static and dynamic movements. Twenty-two healthy young adults (12 females) performed standing, walking, running, and jumping movements while simultaneously collecting pressure (GP MobilData WiFi, GeBioM mbH, Münster, Germany) and force (Kistler^®^) data twice, 10 days apart. Concerning validity, ICC values showed excellent agreement (ICC > 0.75), irrespective of the test condition. Further, the insoles underestimated (mean bias: −4.41 to −37.15%) most of the vGRF variables. Concerning reliability, ICC values for nearly all test conditions also showed excellent agreement, and the SEM was rather low. Lastly, most of the MDC_95%_ values were low (≤5%). The predominantly excellent ICC values for between-devices (i.e., concurrent validity) and between-visits (i.e., test–retest reliability) comparisons suggest that the tested pressure-detecting insoles can be used under field-based conditions for a valid and reliable estimation of relevant vGRF variables during standing, walking, running, and jumping.

## 1. Introduction

The ground reaction force has great importance in sports because many movements (i.e., jumping, throwing, hitting movements) are based on a kinetic chain to achieve high-performance outcomes (i.e., jump height, throwing distance, hitting speed) [1,2,3]. The ground reaction force is divided into vertical and horizontal components, which are mostly measured under laboratory conditions by means of a force-plate [2]. Yet, the vertical component of the ground reaction force, which is of particular interest in various contexts (i.e., clinical gait analysis [4,5], performance analysis in sports [6,7], injury prevention [8,9], development of sports shoes [10]), can also be assessed using pressure-detecting insole systems. In addition, insoles compared to a force-plate have the advantage that they can be used under field-based (e.g., tennis court, soccer field) rather than laboratory conditions and are less expensive and easier to operate [11]. Another virtue of pressure-detecting insole systems is that several consecutive steps can be registered during walking and running [12]. Lastly, the assessment of vertical ground reaction force (vGRF) with insoles is important because the natural movement pattern, as well as the full range of motion, can be maintained, and no fixed mounted, size-limited force-plate needs to be touched accurately. Since pressure is the quotient of force and area, if the area is known, the resulting overall vertical force related to this area can be calculated from the measurement data of the plantar pressure-detecting insoles [13]. However, the question arises to what extent these pressure-detecting insole systems provide valid as well as reliable estimations of the vGRF compared to a force-plate (i.e., the gold standard and criterion device).

So far, different pressure-detecting insole systems using capacitive or resistive technology (e.g., Pedar Mobile system, Novel GmbH, Munich, Germany; Loadsol^®^, Novel Electronics, St. Paul, MN, USA; Insole3, Moticon ReGo AG, Munich, Germany, F-Scan 300E Sport, Tekscan Inc., Boston, MA, USA; WalkinSense^®^, Tomorrow Options Microelectronics, S.A., Sheffield, UK) have been investigated with respect to validity and reliability [14,15,16,17,18,19]. Although most of these systems were shown to be valid and reliable, only a few movements (i.e., standing or walking or running) were examined with a rather small sample size (e.g., *N* = 11 in the study of Cramer et al. [16]) and, in some cases, only one of two quality criteria were considered (e.g., validity only in the study of Stöggl & Martiner [20]). Therefore, the aim of the present study was to investigate static (i.e., standing) as well as dynamic (i.e., walking, running, and jumping) movements in a larger sample (*N* = 22) using a pressure-detecting insole system (i.e., GP MobilData WiFi, GeBioM mbH, Münster, Germany) that has not yet been tested for concurrent validity and test–retest reliability. The scientific and research value of this plantar pressure system has already been demonstrated in a study with twelve nationally ranked tennis players [21]. More precisely, it was investigated how court surface influences foot work and running speed. As a result, it was shown for the first time that changes in direction time, running time, and ground contact time were significantly longer on a clay court compared to a carpet court when performing a tennis-specific shuttle run test. In sum, we hypothesized to find good to excellent agreement (concurrent validity) between the estimated (insoles) and the measured (force-plate) vGRF variables as well as good to excellent consistency (test–retest reliability) of the insoles for the estimated vGRF parameters between two visits.

## 2. Materials and Methods

### 2.1. Participants

Twenty-two healthy young adults of both sexes took part in the study (Table 1). Their age ranged from 20 to 35 years. All participants were informed about the contents of the respective study and gave their written consent to participate. The human ethics committee at the University of Duisburg-Essen, Faculty of Educational Sciences, approved the study protocol (approval number: TM_02.03.22):

### 2.2. Assessment of Insole Data

**Figure 1:**
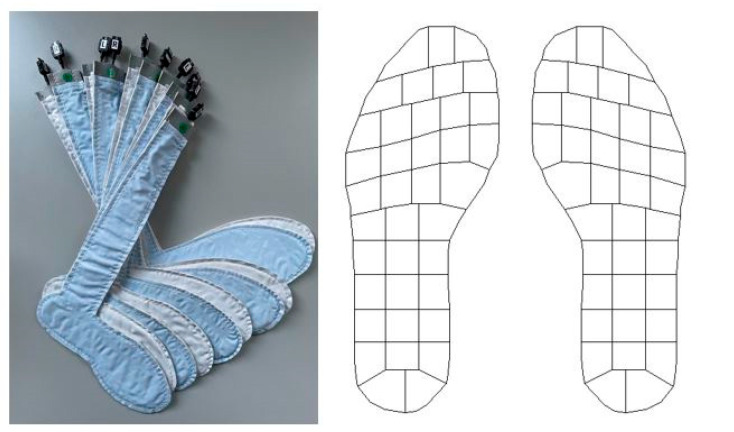
The plantar pressure-detecting insole system (i.e., GP MobilData WiFi, GeBioM mbH, Münster, Germany) and the layout of the sensors.

Flexible instrumented insoles of the GP MobilData WiFi System (GeBioM mbH, Münster, Germany) were used to record plantar pressure data at a sampling rate of 200 Hz (Figure 1). One set of insoles consists of five pairs with different sizes (i.e., EU 37–38, EU 39–40, EU 41–42, EU 43–44, EU 45–46). Depending on the shoe size, each sole consists of 40–64 equally sized sensors (diameter: 8 mm). The obtained data were sent to a laptop via a wireless signal using GP Manager V7 Software (GeBioM mbH, Münster, Germany). The instrumented insoles were placed over the conventional insoles of the individual shoes. The shoes were regularly worn running shoes, and the participants were asked to wear the same shoes on both visits. The system allows exporting of different parameters, e.g., maximum/mean pressure, force values, contact areas, and focus areas. In addition, the foot zones that are examined can be adapted individually depending on the research question (e.g., whole foot, forefoot, rearfoot, or each metatarsal, separately).

### 2.3. Assessment of Force-Plate Data

A three-dimensional force-plate (dimensions: 600 mm × 400 mm × 100 mm, Kistler [type 9281b], Winterthur, Switzerland) was used as gold standard and criterion device to record vGRF (sampling rate: 2,000 Hz). The force-plate was connected to a personal computer, and the Vicon Nexus software 2 (VICON, Oxford, UK) allowed quantification of vGRF variables.

### 2.4. Procedures

**Figure 2:**
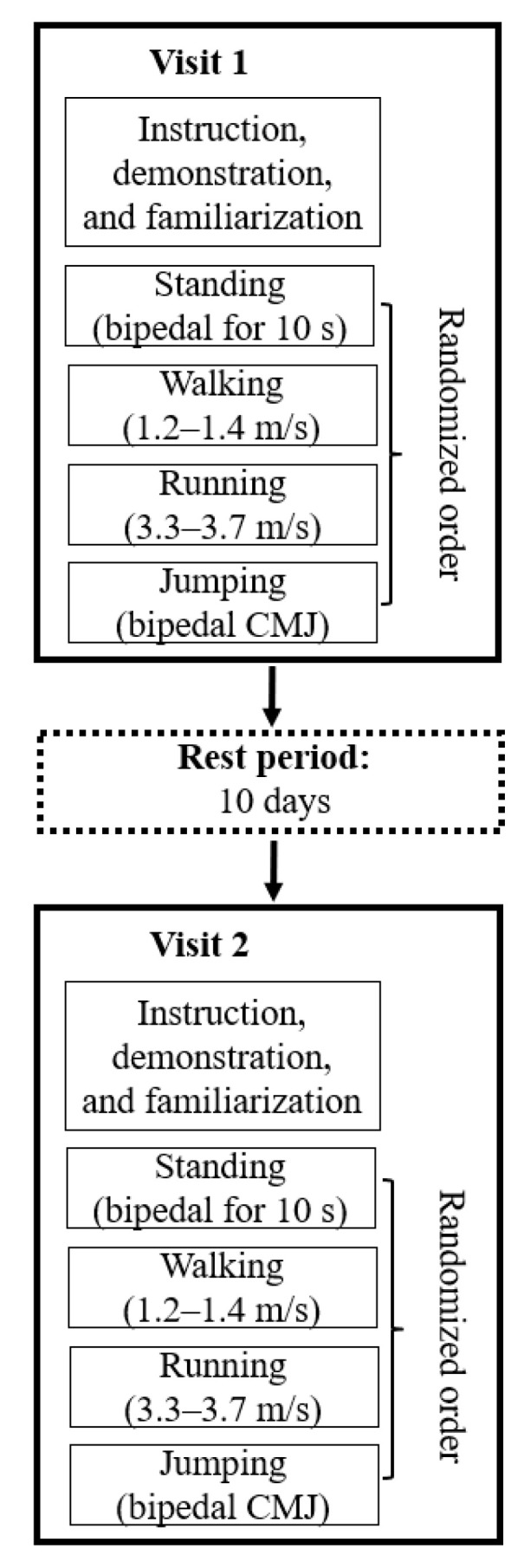
Schematic diagram of the study design and testing procedure. CMJ = countermovement jump.

At the beginning of a test session, the participants received a verbal briefing of the experimental design, a practical demonstration of the different test conditions, and were familiarized with the pressure-detecting insoles. Familiarization lasted 10 min and included standing, walking, running, and jumping exercises (Figure 2). Each participant used the appropriate insole for their shoe size and their own sports shoes. Subsequently, the following four test conditions were performed in a randomized order: (a) bipedal stance for 10 s, (b) walking (1.2–1.4 m/s), (c) running (3.3–3.7 m/s), and (d) bipedal countermovement jump with the hands fixed to the waist. To achieve a better comparability of the results, walking and running speeds were adapted from previous studies [16,18]. In order to reach the predetermined target speed during conditions (b) and (c), practice trials were performed. Two photoelectric sensors (WITTY, Microgate Srl, Bolzano, Italy) were placed at equal distances (2.5 m) before and behind the force-plate at knee height and measured speed. For each test condition, five successful trials were recorded. During walking and running, a trial was considered successful if the participant reached the predetermined target speed and hit the force-plate with the right foot without having to adjust speed or stride length. Between trials and test conditions, 30 s and 60 s rest periods, respectively, were provided, and each participant repeated the aforementioned procedure after 10 days.

### 2.5. Data Processing

The analysis of the insole data was performed using MATLAB software version R2017a (The MathWorks Inc., Natick, MA, USA). The time stamp from the force-plate data was used to determine the equivalent step during walking and running (always the second step of the right lower limb due to the distance chosen and visual verification) from the insole system data. Different variables were calculated for each condition. During the bipedal stance, mass was calculated. For walking, the first and second local maximum (peak 1 and peak 2), the local valley between the peaks, the impulse (N∙s), and the contact time (s) were calculated. For running, the maximum peak (peak max), the impulse (N∙s), and the contact time (s) were determined. For jumping, the maximum vGRF during take-off (propulsive force) and landing (landing force), as well as jump height (cm), were computed. Jump height was calculated using the “flight-time” method. The mean of the successful trials was used to perform the statistical analysis. All force variables were measured in Newton (N). For the bipedal stance and jump, the vGRF was calculated from the sum of the two insoles (right and left).

### 2.6. Statistical Analyses

Assumptions of normality (Shapiro–Wilk Test) and homogeneity of variance/sphericity (Mauchly Test) were checked and met prior to the application of statistical analyses. Descriptive data are reported as group mean values and standard deviations (SD). Concurrent validity between the force-plate (i.e., gold standard and criterion device) and the insole data was quantified using the mean values of visits 1 and 2. Specifically, the intraclass correlation coefficient (ICC_3,k_) for mean-rating (k = 2, two-way mixed effects model between subject and device) with a 95% confidence interval (CI) and the percent bias (i.e., mean difference of measurements between both devices as a proportion of the force-plate values) were calculated. According to Fleiss [22], ICC ≥ 0.75 was considered “excellent”, 0.40 ≤ ICC < 0.75 was considered “moderate-to-good”, and ICC < 0.40 was considered “poor”. Further, Bland–Altman graphics were created to illustrate the agreement between force-plate and insole data, i.e., the differences were plotted against the mean between both devices. It is recommended that 95% of the data points should be within the limits of agreement (i.e., mean value ± 1.96 SD).

Test–retest reliability was quantified by comparing the data from visits 1 and 2 for each measuring device separately. Precisely, relative reliability was determined by calculating the ICC_3,2_ (two-way mixed-effects model between subjects and visits) with a 95% CI. Absolute reliability was assessed using the standard error of measurement (SEM). Further, we calculated the minimal detectable change (MDC_95%_), which indicates the size of a measurement difference in a repeated measurement at which a measurement error can be excluded and a real change is present. All statistical analyses were performed using SPSS software (version 27.0, IBM Corporation, Armonk, NY, USA).

## 3. Results

### Concurrent Validity

**Figure 3:**
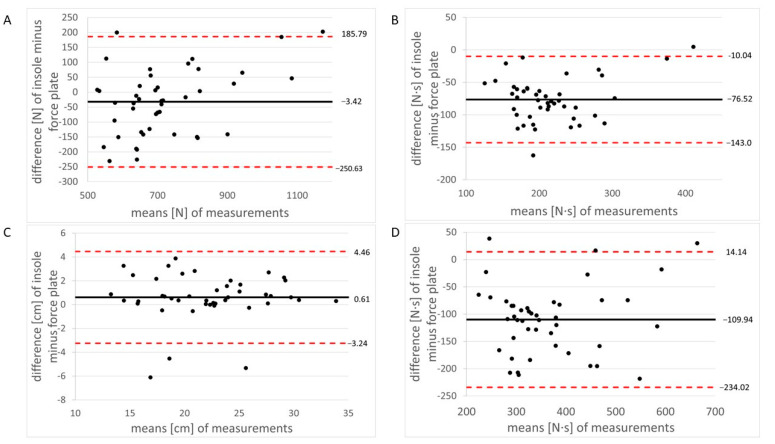
Bland–Altman plots for the comparison of the pressure-detecting insoles with the force-plate (i.e., the gold standard and criterion device) during (**A**) standing (i.e., mass), (**B**) walking (i.e., impulse), (**C**) running (i.e., impulse), and (**D**) jumping (i.e., jump height). The mean of the differences (insole minus force-plate) is indicated by the solid black line, and 95% confidence intervals are indicated by dashed red lines.

Table 2 shows the descriptive data and concurrent validity of the pressure-detecting insoles compared to the force-plate (i.e., the gold standard and criterion device). Irrespective of the test condition, we found “excellent” ICC values > 0.75. Furthermore, the mean bias values demonstrate that, except for contact time during walking and running and for jump height during jumping, most of the registered data were underestimated by the insoles compared to the force-plate. In addition, exemplary Bland–Altman plots revealed that most of the data points lie within the limits of agreement (Figure 3A–D). Specifically, only 2 out of 22 points (9.1%), 3 out of 22 points (13.6%), 2 out of 22 points (9.1%), and 3 out of 22 points (13.6%) were outside the limits during standing (i.e., mass), walking (i.e., impulse), running (i.e., impulse), and jumping (i.e., jump height), respectively.

Descriptive data and test–retest reliability of the pressure-detecting insoles are presented in Table 3. In terms of relative reliability, we detected “excellent” ICC values for all but one (i.e., a “moderate-to-good” ICC value of 0.74 for contact time during walking) test condition. Concerning absolute reliability, the absolute SEM value was 45.16 for standing and ranged from 0.03–40.12 for walking, from 0.01–57.04 for running, and from 2.21–227.34 for jumping. This corresponds to values of 6–7%, 4–9%, 4–6%, and 8–10% for standing, walking, running, and jumping, respectively. In addition, the MDC_95%_ value was 2.65% for standing and ranged from 2.44–65.73% for walking, from 1.70–112.00% for running, and from 1.82–18.41% for jumping. Table 4 illustrates the descriptive data and test–retest reliability of the force-plate. Again, the detected ICC values were “excellent” for all but one (i.e., a “moderate-to-good” ICC value of 0.74 for contact time during walking) test condition. The absolute SEM value was 21.53 for standing and ranged from 0.03–32.88 for walking, from 0.01–46.35 for running, and from 1.09–148.00 for jumping. This equals values of 3%, 4–6%, 3–8%, and 5–9% for standing, walking, running, and jumping, respectively. Additionally, the MDC_95%_ value was 1.75% for standing and ranged from 1.80–70.00% for walking, from 1.08–88.0% for running, and from 1.14–13.09% for jumping.

## 4. Discussion

To our knowledge, the present study investigated concurrent validity and test–retest reliability of a pressure-detecting insole system (i.e., GP MobilData WiFi, GeBioM mbH, Münster, Germany) for the assessment of vGRF variables during static (i.e., standing) and dynamic (i.e., walking, running, and jumping) movements in healthy young female and male adults for the first time. The main results can be summarized as follows: (1) irrespective of test condition, “excellent” agreement (i.e., ICC values) was detected between the estimated (insole) and the measured (force-plate) vGRF variables; (2) most of the vGRF parameters were underestimated (−4.41 to −37.15%) by the insoles compared to the force-plate; (3) the majority of data points (i.e., 19–20 out of 22 values) lie within the limits of agreement; (4) in nearly all vGRF variables, “moderate-to-good” to “excellent” agreement (i.e., ICC values) was detected between visit 1 and 2 for the pressure-detecting insole system; (5) for most of the vGRF parameters, the minimum amount of change needed to identify clinically relevant effects in repeated insole measurements was rather low (≤5%).

### 4.1. Concurrent Validity of the Pressure-Detecting Insoles Compared to the Force-Plate

In line with our initial hypothesis stating good to excellent agreement between the estimated (insoles) and the measured (force-plate) vGRF variables, the analysis revealed “excellent” values (ICC: > 0.75) for all outcomes during standing (ICC: 0.86), walking (ICC_1.2–1.4 m/s_: 082–0.98), running (ICC_3.3–3.7 m/s_: 0.87–0.91), and jumping (ICC: 0.82–0.96). Regarding walking and running, our results correspond with those from previous studies [16,18,19] that investigated the validity of pressure-detecting insole systems and reported excellent agreement with a force-plate (i.e., criterion device and the gold standard). For example, Renner et al. [18] investigated recreationally active adults between the ages of 18–30 years and reported “excellent” values for walking (ICC_1.3 m/s_: 0.80–0.97) and running (ICC_3.0 m/s_: 0.86–0.96, ICC_3.5 m/s_: 0.83–0.97). Further, Cramer et al. [16] studied healthy adults aged 33.1 ± 16.7 years and observed “excellent” values for slow walking (ICC_0.8–1.0 m/s_: 0.98–0.99), moderate-paced walking (ICC_1.2–1.4 m/s_: 0.94–0.98), and running (ICC_3.3–3.7 m/s_: 0.94). With respect to standing and jumping, Stöggl and Martiner [20] performed a validation of an insole system using a force-plate in young, healthy adults (age: 31 ± 10 years). Although only Pearson’s product-moment correlation coefficients (*r*-values) were calculated, the *r*-values proved to be “high” for both test conditions (*r* = 0.77–0.83). The Bland–Altman plots (Figure 3A–D) supported our findings concerning excellent validity as almost all data points were within the agreement limits, which is consistent with former studies [16,18]. In addition, we observed—based on the mean bias values—that most of the registered data were underestimated by the pressure-detecting insole system compared to the force-plate, i.e., standing: −4.41%; walking: −26.17 to −37.15%; running: −29.41 to −30.15%; jumping: −23.30 to −24.73%. Both Renner et al. [18] for walking at 1.3 m/s and running at 3.0 m/s and 3.5 m/s as well as Cramer et al. [16] for walking at 0.8–1.0 m/s and 1.2–1.4 m/s and running at 3.3–3.7 m/s also reported an underestimation by the insoles. However, contrary to Stöggl and Martiner [20] and Nagahara and Morin [23], we did not observe an increase in mean bias despite increasing force values from walking (513.84–719.08 N) over running (1229.06 N) to jumping (1186.63–2200.74 N). Accordingly, the tested GP MobilData WiFi insole system seems to record vertical ground reaction forces correctly during both slow (walking) and fast (running, jumping) movements. One reason for the underestimation of the measured values could be the dampening effect of the used footwear (i.e., running shoes with soft conventional insoles). In this regard, Kalpen and Seitz [24] stated that the forces between ground and shoe are different from those between foot and shoe. In addition, the pressure-detecting insoles are flexible and may thus have allowed a deformation due to foot morphology and/or the applied dynamic movements (i.e., walking, running, and jumping). Therefore, the pressure-detecting sensors were no longer perfectly vertical, resulting in imprecise recordings of the vGRF [25].

From a practical perspective, our finding of “excellent” validity indicates that the tested GP MobilData WiFi insole system can be used as a cost-effective alternative to a force-plate for assessing relevant vGRF variables during static (i.e., standing) and dynamic (i.e., walking, running, and jumping) movements in healthy young female and male adults. However, when analysing the registered plantar pressure data, the underestimation of the estimated force values should be considered.

### 4.2. Test–Retest Reliability of the Pressure-Detecting Insoles

In accordance with our initial hypothesis stating good to excellent consistency of the estimated (insoles) vGRF variables between visits 1 and 2, the analysis showed “excellent” values for all outcomes during standing (ICC: 0.94), running (ICC_3.3–3.7 m/s_: 0.85–0.98), and jumping (ICC: 0.81–0.91) and “moderate-to-good” to “excellent” values for walking (ICC_1.2–1.4 m/s_: 0.74–0.97). Renner et al. [18] also investigated test–retest reliability and likewise reported “excellent” values for walking (ICC_1.3 m/s_: 0.90–0.96) and running (ICC_3.0 m/s_: 0.85–0.91, ICC_3.5 m/s_: 0.88–0.91). Moreover, Cramer et al. [16] studied test–retest reliability and also observed “excellent” values for slow walking (ICC_0.8–1.0 m/s_: 0.99), moderate-paced walking (ICC_1.2–1.4 m/s_: 0.98–0.99), and running (ICC_3.3–3.7 m/s_: 0.97–0.98). In the present study, the absolute SEM values amounted to 45.16 for standing and ranged from 0.03–40.12 for walking, from 0.01–57.04 for running, and from 2.21–227.34 for jumping. This corresponds to relatively low percentage values of 6–7% (standing), 4–9% (walking), 4–6% (running), and 8–10% (jumping), again indicating good test–retest reliability.

Further, the detected MDC_95%_ values (i.e., the minimum amount of change needed to identify clinically relevant effects in repeated insole measurements) were rather low (≤5%) in most of the vGRF variables. Thus, a change in plantar pressure-derived vGRF data exceeding these values seems to be a true response, and a clinician or therapist can be 95% confident that beyond measurement error, a true change has occurred. In addition, comparatively high MDC_95%_ values were found for contact time during walking (65.73%) and running (112.00%), as well as jump height during jumping (18.41%). Regarding walking and running, this finding might be explained by contact time’s dependence on cadence and walking/running speed [26]. More specifically, it may be hypothesized that between the two measurements, the same participants chose different speeds and cadences within the predetermined target speed interval of 1.2–1.4 m/s for walking and of 3.3–3.7 m/s for running. In terms of jumping, it seems most likely that the same participants achieved different jump heights on visit 1 compared to visit 2. This indicates a certain difficulty in repeatedly generating maximal vertical jump heights.

For practitioners, our finding of “moderate-to-good” to “excellent” test–retest reliability indicates that the tested GP MobilData WiFi insole system can be used as a reliable device for assessing relevant vGRF variables. For all variables, except for the contact time during walking and running, as well as for the jump height during jumping, the MDC_95%_ values can be used as a threshold for the detection of clinically relevant changes between repeated insole measurements.

The present study has some strengths and limitations. In terms of the study values, we have investigated both concurrent validity and test–retest reliability for relevant daily and athletic physical activities (i.e., standing, walking, running, and jumping movements) in a relatively large sample of *N* = 22 healthy young female and male adults for the first time. We mostly found excellent agreement between the pressure-detecting insoles and the force-plate (i.e., concurrent validity) as well as between repeated measurements (i.e., test–retest reliability), regardless of the movement considered. The obtained results are important for further research questions, such as the investigation of physical training programmes to change plantar pressure patterns. Regarding study limitations, we used insoles with shoe sizes EU 37–46 only. Consequently, no conclusions can be drawn regarding individuals with smaller or larger shoe sizes. Secondly, unlike force-plates, pressure-detecting insoles are known to take time for restitution after deformation (e.g., heel-down during walking or running), and the foot is vertically aligned a few milliseconds before toe-off (e.g., during jumping). Thus, the measurement of vGRF via insoles is limited in both situations. Thirdly, differences in force values between visits cannot solely be explained by the respective measuring system because we used a predetermined target speed interval for walking (1.2–1.4 m/s) and running (3.3–3.7 m/s) but not a fixed target speed value. Therefore, the same participant may have been walking/running at the lower limit at visit 1 and at the upper limit at visit 2, resulting in different force values. Further, participants were asked to jump as high as possible during the countermovement jump. However, they may have had difficulties replicating the individual maximal jumping height between the two visits, which would eventually have resulted in discrepancies between force values. Fourthly, only young adults were studied; thus, no comment can be made regarding younger (i.e., children and adolescents) and older (i.e., seniors) individuals.

## 5. Conclusions

Our findings of “excellent” ICC values and few data points outside the agreement limits indicate that the tested GP MobilData WiFi insole system is a valid alternative to a force-plate for the assessment of vGRF variables during static (i.e., standing) and dynamic (i.e., walking, running, and jumping) movements in young and healthy female and male adults. However, when analysing the estimated (insole) vGRF parameters, care should be taken as they underestimate the majority of the measured (force-plate) vGRF variables. Further, our results of “moderate-to-good” to “excellent” ICC values and rather low SEM values suggest that the tested insole system is a reliable alternative to a force-plate for assessing vGRF parameters under field-based conditions, such as on the tennis court or soccer field. Most MDC_95%_ values were rather low and can be used as a threshold to determine clinically relevant changes between repeated insole measurements.

## Figures and Tables

**Table 1:** Characteristics of the study participants (*N* = 22).

Characteristic	Value
Age [years]	27.5 ± 9.2
Sex [female/male]	12/10
Body height [cm]	178.6 ± 3.7
Body mass [kg]	74.9 ± 13.6
Shoe size [EU]	42.0 ± 2.8

Data represent means ± standard deviations.

**Table 2:** Descriptive data and concurrent validity of the pressure-detecting insoles (i.e., GP MobilData WiFi, GeBioM mbH) compared to the force-plate (i.e., Kistler^®^, type 9281b).

Test Condition	vGRF Variable	Force-Plate Mean ± SD	Insoles Mean ± SD	ICC_3,2_ (95% CI)	Mean Bias [%]
Standing	Mass [N]	734.43 ± 133.54	702.01 ± 177.13	0.86 (0.74–0.92)	−4.41
Walking	Peak 1 [N]	845.10 ± 150.30	719.08 ± 187.08	0.82 (0.67–0.90)	−14.91
	Peak 2 [N]	817.60 ± 148.51	513.84 ± 200.29	0.87 (0.76–0.93)	−37.15
	Valley [N]	510.66 ± 111.57	376.12 ± 142.09	0.85 (0.72–0.92)	−26.35
	Impulse [N∙s]	420.15 ± 103.43	310.21 ± 110.47	0.90 (0.82–0.95)	−26.17
	Contact time [s]	0.70 ± 0.06	0.72 ± 0.06	0.98 (0.95–0.99)	1.85
Running	Peak max [N]	1741.02 ± 299.17	1229.06 ± 368.19	0.91 (0.84–0.95)	−29.41
	Impulse [N∙s]	253.77 ± 55.25	177.25 ± 63.04	0.91 (0.84–0.95)	−30.15
	Contact time [s]	0.25 ± 0.02	0.25 ± 0.03	0.87 (0.77–0.93)	0.97
Jumping	Propulsive force [N]	1576.59 ± 308.44	1186.63 ± 303.11	0.82 (0.67–0.90)	−24.73
	Landing force [N]	2869.35 ± 613.12	2200.74 ± 716.91	0.95 (0.90–0.97)	−23.30
	Jump height [cm]	22.39 ± 5.03	21.78 ± 4.99	0.96 (0.93–0.98)	2.80

ICC_3,k_ = intraclass correlation coefficient; CI = confidence interval; SD = standard deviation; vGRF = vertical ground reaction force; LoA = limits of agreement.

**Table 3:** Descriptive data and test–retest reliability of the pressure-detecting insoles (i.e., GP MobilData WiFi, GeBioM mbH).

Test Condition	vGRF Variable	Insoles: Visit 1 Mean ± SD	Insoles: Visit 2 Mean ± SD	ICC_3,2_ (95% CI)	SEM *	MDC_95%_ [%]
Standing	Mass [N]	716.35 ± 198.65	687.68 ± 156.05	0.94 (0.84–0.97)	45.16	2.65
Walking	Peak 1 [N]	742.33 ± 197.38	695.83 ± 177.69	0.95 (0.89–0.98)	40.12	2.44
	Peak 2 [N]	524.97 ± 215.85	502.71 ± 187.85	0.97 (0.93–0.99)	34.69	3.17
	Valley [N]	382.73 ± 151.80	369.52 ± 134.94	0.95 (0.87–0.98)	33.32	4.25
	Impulse [N∙s]	318.43 ± 118.20	301.99 ± 104.28	0.97 (0.93–0.99)	20.67	4.06
	Contact time [s]	0.72 ± 0.06	0.71 ± 0.06	0.74 (0.36–0.89)	0.03	65.73
Running	Peak max [N]	1253.11 ± 383.07	1205.02 ± 360.05	0.98 (0.94–0.99)	57.04	1.70
	Impulse [N∙s]	183.05 ± 67.80	171.45 ± 58.90	0.97 (0.93–0.99)	10.55	5.08
	Contact time [s]	0.25 ± 0.02	0.25 ± 0.03	0.85 (0.65–0.94)	0.01	112.00
Jumping	Propulsive force [N]	1214.55 ± 316.83	1158.71 ± 293.45	0.89 (0.74–0.96)	99.15	2.32
	Landing force [N]	2391.45 ± 822.78	2192.24 ± 691.49	0.91 (0.78–0.96)	227.34	1.82
	Jump height [cm]	23.31 ± 4.31	21.46 ± 5.60	0.81 (0.53–0.92)	2.21	18.41

* Absolute SEM values are provided, and therefore the unit corresponds to the respective vGRF variable. ICC_3,k_ = intraclass correlation coefficient; CI = confidence interval; MDC_95%_ = minimal detectable change; SD = standard deviation; SEM = standard error of measurement; vGRF = vertical ground reaction force.

**Table 4:** Descriptive data and test–retest reliability of the force-plate (i.e., Kistler^®^, type 9281b).

Test Condition	vGRF Variable	Force-Plate: Visit 1 Mean ± SD	Force-Plate: Visit 2 Mean ± SD	ICC_3,2_ (95% CI)	SEM *	MDC_95%_ [%]
Standing	Mass [N]	728.66 ± 143.48	740.21 ± 125.93	0.97 (0.94–0.99)	21.53	1.75
Walking	Peak 1 [N]	837.59 ± 162.70	852.60 ± 140.24	0.96 (0.90–0.98)	30.06	1.80
	Peak 2 [N]	806.25 ± 152.49	828.94 ± 147.10	0.95 (0.83–0.97)	32.88	1.94
	Valley [N]	506.95 ± 120.87	514.37 ± 104.16	0.93 (0.83–0.97)	29.31	2.93
	Impulse [N∙s]	415.64 ± 111.84	424.66 ± 96.72	0.94 (0.85–0.98)	25.55	3.33
	Contact time [s]	0.70 ± 0.06	0.70 ± 0.06	0.74 (0.37–0.89)	0.03	70.00
Running	Peak max [N]	1752.72 ± 324.65	1729.32 ± 278.53	0.98 (0.94–0.99)	46.35	1.08
	Impulse [N∙s]	253.93 ± 60.58	253.62 ± 50.79	0.88 (0.71–0.95)	19.14	4.78
	Contact time [s]	0.25 ± 0.03	0.25 ± 0.02	0.94 (0.85–0.97)	0.01	88.00
Jumping	Propulsive force [N]	1568.84 ± 293.52	1584.35 ± 328.66	0.95 (0.87–0.98)	138.11	2.06
	Landing force [N]	3007.96 ± 655.82	2913.41 ± 629.10	0.95 (0.88–0.98)	148.00	1.14
	Jump height [cm]	23.13 ± 4.26	21.01 ± 5.30	0.80 (0.52–0.92)	1.09	13.09

* Absolute SEM values are provided, and therefore the unit corresponds to the respective vGRF variable. ICC_3,k_ = intraclass correlation coefficient; CI = confidence interval; MDC_95%_ = minimal detectable change; SD = standard deviation; SEM = standard error of measurement; vGRF = vertical ground reaction force.

## Data Availability

The data presented in this study are available on request from the corresponding author. The data are not publicly available due to ethical restrictions.
